# Genome-wide identification and expression profiling of the C2H2-type zinc finger protein genes in the silkworm *Bombyx mori*

**DOI:** 10.7717/peerj.7222

**Published:** 2019-07-05

**Authors:** SongYuan Wu, Xiaoling Tong, ChunLin Li, KunPeng Lu, Duan Tan, Hai Hu, Huai Liu, FangYin Dai

**Affiliations:** 1 State Key Laboratory of Silkworm Genome Biology, Key Laboratory of Sericultural Biology and Genetic Breeding, Ministry of Agriculture, College of Biotechnology, Southwest University, Chong Qing, China; 2 College of Plant Protection, Southwest University, Chong Qing, China

**Keywords:** C2H2-ZF, Silkworm, Characterization, Expression pattern, Functional association

## Abstract

Cys2-His2 zinc finger (C2H2-ZF) proteins comprise the largest class of putative eukaryotic transcription factors. The zinc finger motif array is highly divergent, indicating that most proteins will have distinctive binding sites and perform different functions. However, the binding sites and functions of the majority of C2H2-ZF proteins remain unknown. In this study, we identified 327 C2H2-ZF protein genes in the silkworm, 290 in the monarch butterfly, 243 in the fruit fly, 107 in elegans, 673 in mouse, and 1,082 in human. The C2H2-ZF protein genes of the silkworm were classified into three main grouping clades according to a phylogenetic classification, and 312 of these genes could be mapped onto 27 chromosomes. Most silkworm C2H2-ZF protein genes exhibited specific expression in larval tissues. Furthermore, several C2H2-ZF protein genes had sex-specific expression during metamorphosis. In addition, we found that some C2H2-ZF protein genes are involved in metamorphosis and female reproduction by using expression clustering and gene annotation analysis. Among them, five genes were selected, *BGIBMGA002091* (*CTCF*), *BGIBMGA006492* (*fru*), *BGIBMGA006230* (*wor*), *BGIBMGA004640* (*lola*), and *BIGBMGA004569*, for quantitative real-time PCR analysis from larvae to adult ovaries. The results showed that the five genes had different expression patterns in ovaries, among which *BGIBMGA002091* (*CTCF*) gene expression level was the highest, and its expression level increased rapidly in late pupae and adult stages. These findings provide a basis for further investigation of the functions of C2H2-ZF protein genes in the silkworm, and the results offer clues for further research into the development of metamorphosis and female reproduction in the silkworm.

## Introduction

Transcription factors (TFs) play an important role in regulating gene expression (Latchman, 1990). A TF is characterized by the DNA-binding domain; some TFs also contain other effector domains. TFs are involved in multiple biological processes, including development (Sommer et al., 1992), metabolism (Krebs et al., 2014), apoptosis (Ma et al., 2016), autophagy, (Chauhan et al., 2013), and stemness maintenance (Lai et al., 2014). The Cys2-His2 zinc finger proteins (C2H2-ZF) number more than 700 and are the largest family of TFs found in humans (Vaquerizas et al., 2009; Weirauch & Hughes, 2011). In addition, zinc finger array and putative DNA-binding sequences are significantly differentiated, indicating that the functions of C2H2-ZF are diverse. The C2H2-ZF motif is composed of Cys-X_2,4_-Cys-X_12_-His-X_3,4,5_-His, and the motif interacts with zinc ions and folds into a finger-like structure. This finger-like structure is composed of α-helix and antiparallel β-sheet (Klug & Schwabe, 1995; McCarty et al., 2003).

In general, the binding sequence of the C2H2-ZF motif is according to the amino acid residue sequence of its α-helical component. Regarding the first amino acid residue of the α-helical region in the C2H2-ZF domain as position 1, the four positions −1, 2, 3, and 6 are the key amino acids for recognition and binding of DNA via interaction with the hydrogen donors and acceptors exposed in the major groove. The −1, 3, and 6 amino acid residues bind to base 3, base 2, and base 1, respectively, in the sense sequence of DNA, and the amino acid residue of position 2 binds to the fourth base of the antisense sequence (Klug, 2010; Wolfe, Nekludova & Pabo, 2000) (Fig. S1). Many TFs contain a group of tandem zinc fingers, and the prediction of their recognition sequence often depends on single zinc finger. However, the recognition sequences of individual zinc finger proteins may be modified due to adjacent zinc fingers. Therefore, it is difficult to analyze the recognition sequences of tandem zinc finger domains (Lam et al., 2011; Ramirez et al., 2008). In recent years, some bioinformatic analytical approaches have been made, along with chromatin immunoprecipitation followed by sequencing (ChIp-seq) data for tandem zinc finger proteins (Gupta et al., 2014; Hamzeh-Mivehroud et al., 2015; Jayakanthan et al., 2009; Persikov & Singh, 2014; Sandelin et al., 2004; Sander et al., 2009; Wolfe et al., 1999; Yang et al., 2013). These methods provide important references for studying the molecular mechanism and function of zinc finger TFs. Nonetheless, the zinc finger family is numerous, and there is significant divergence among species. Zinc finger transcript factors participate in multiple biological processes by transcript regulation, and thus clarifying the function and molecular mechanism of zinc fingers is complicated. Therefore, further studies of the zinc finger protein family are important for understanding the function and evolution of TFs.

The silkworm *Bombyx mori* was domesticated from the Chinese wild silkworm *B. mandarina* over 5,000 years ago (Sun et al., 2012). The silkworm has long been an economically important insect due to the production of silk. From the 19th century, the silkworm became a model for scientific discovery in microbiology, physiology, and genetics (Goldsmith, Shimada & Abe, 2005). Silkworms, which have undergone a long period of domestication and artificial selection, have a clearly understood genetic background. In addition, *B. mori* became a representative organism of Lepidoptera for basic research following the sequencing of the whole genome (Meng, Zhu & Chen, 2017). In this study, we identified C2H2-ZF proteins in the zinc finger gene family by genome-wide analysis, and we performed sequence and microarray analyses. Our findings should provide a basis for better understanding of the C2H2-ZF protein families, and the results may provide important clues for further research concerning the functions of TFs in the silkworm and Lepidoptera.

## Materials and Methods

### Genome-wide identification of the genes encoding C2H2-ZF proteins

The whole-genome protein sequences of *B. mori* were downloaded from the silkworm genome databases Kaikobase (http://sgp.dna.affrc.go.jp/KAIKObase/) (Shimomura et al., 2009). The hidden Markov model (HMM) profile of the C2H2-ZF (PF00096) was downloaded from the Pfam database (https://pfam.xfam.org) (Sonnhammer, Eddy & Durbin, 1997). HMMER 3.0 software was downloaded from http://www.hmmer.org, and the hmmsearch program was used to search for C2H2 zinc finger proteins in the silkworm genome, and the expected value was set to be less than 0.01 (Finn, Clements & Eddy, 2011). The candidate proteins containing C2H2 zinc finger domains were identified on the SMART website (http://smart.embl-heidelberg.de/) (Ponting et al., 1999). The protein sequences of *Danaus plexippus* were downloaded from MonarchBase (http://monarchbase.umassmed.edu) (Zhan & Reppert, 2013), the proteins of *Drosophila melanogaster* were downloaded from flybase (http://flybase.org) (Gramates et al., 2017), the protein sequences of *Caenorhabditis elegans* were downloaded from Wormbase (http://www.wormbase.org) and the protein sequences of *Homo sapiens* and *Mus musculus* were downloaded from NCBI (https://www.ncbi.nlm.nih.gov). BLASTP searches against the non-redundant (nr) protein database were performed to annotate the C2H2-type zinc finger protein genes.

### Chromosomal distribution and domain analysis of the C2H2-ZF protein genes

The chromosomal sizes and gene locations were obtained from KAIKObase. The MapChart 2.32 software was used to draw the chromosomal distribution map (Voorrips, 2002). The analysis of conservative domains used the hmmscan program (Finn, Clements & Eddy, 2011). The well-characterized protein domain families with high-quality alignment data, Pfam-A (Sonnhammer, Eddy & Durbin, 1997), were downloaded from NCBI. The proteins of *B. mori*, *Danaus plexippus*, *Drosophila melanogaster, C. elegans, M. musculus*, and *H. sapiens* were searched against Pfam-A to annotate the domains, and the parameters are defaulted. The complete amino acid sequences of the identified BmZNF proteins were imported into MEGA7 (Kumar, Stecher & Tamura, 2016), and the MUSCLE program carried out multiple sequence alignments (Edgar, 2004). The alignment result was then used to build an unrooted phylogenetic tree by the neighbor-joining method with a bootstrap of 2,000 replicates (Saitou & Nei, 1987).

### Expression profiling analysis

The gene expression data were downloaded from a *B. mori* microarray data (Xia et al., 2007). The multiple larval tissues examined from the third day of the fifth instar included testis, ovary, head, integument, fat body, midgut, hemocyte, Malpighian tubules, anterior/middle silk gland, and posterior silk gland. In addition, the 19 developmental time points, from later larval to adult stages, included day 4 of the fifth instar (5th4d), 5th5d, 5th6d, 5th7d, the beginning of the wandering stage for spinning (W0), 12 h after wandering (W12h), W24h, W36h, W48h (spinning finished), beginning of pupation (P0), 1 day after pupation (P1), P2, P3, P4, P5, P6, P7, P8, and day 1 of the adult moth. In the microarray data analysis, if the signal intensity of the expression of a gene exceeded 400 units at a time point, this gene was considered to be expressed at that time point. In microarray analysis of metamorphosis, form 5th4d to adult, level of expression of genes in the silkworm larvae on day 3 of the fifth instar (5th3d) was used as a control. The ratios of the expression levels of genes were calculated by comparing gene expression with that in the 5th3d control. The expression array figures were drawn with the Pheatmap program of the R package (R Development Core Team, 2015).

### Ovary dissect and qRT–PCR

The silkworm wild-type strain Dazao were obtained from the Silkworm Gene Bank of Southwest University, Chong Qing, China. The ovary of silkworm was removed using 0.7% normal saline solution, and then stored at −80 °C until RNA extraction was performed. Total RNA was isolated from the ovary using E.Z.N.A.^®^ MicroElute Total RNA Kit (Omega Bio-tek, Norcross, GA, USA) according to the manufacturer’s protocol. The primers that were used for qRT-PCR were list in Table S1. qRT-PCR was performed using a CFX96™ Real-Time PCR Detection System (Bio-Rad, Hercules, CA, USA) with SYBR Green qRT-PCR Mix (Bio-Rad, Hercules, CA, USA). The cycling parameters were as follows: 95 °C for 4 min followed by 40 cycles of 95 °C for 10 s and annealing for 30 s. The relative expression levels were analyzed using the classical *R* = 2^−ΔΔCt^ method. The gene for eukaryotic translation initiation factor 4A (NP_001037376.1) of silkworm was used as an internal control to normalize for equal sample loading.

## Results

### Identification and chromosomal distribution of the BmZNF family

**Figure 1:**
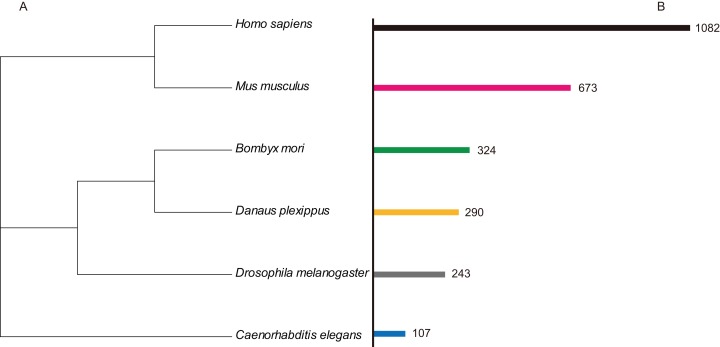
The numbers of ZNF protein gene family member in the different species. (A) Indicated the species tree. (B) Indicated the C2H2-type zinc finger (ZNF) protein genes numbers in different species.

In order to identify C2H2-ZF protein genes in *B. mori*, the hmmsearch program in the HMMER3.0 package (Finn, Clements & Eddy, 2011) (http://www.hmmer.org) was used to search the silkworm comprehensive genetic data sets (Suetsugu et al., 2013), and HMM profiles data were used for the C2H2-ZF motif (PF00096). The output data were confirmed by SMART (Ponting et al., 1999) (Simple Modular Architecture Research Tool, http://smart.embl-heidelberg.de) and manually modified. As a result, 327 unique silkworm C2H2 zinc finger protein genes were obtained, and these were named in an abbreviated format as BmZNF (*B. mori* zinc fingers C2H2-type) based on the principles for the nomenclature of C2H2 zinc finger proteins described by the HUGO Gene Nomenclature Committee (HGNC) (Povey et al., 2001). In addition, all of the *BmZNF* genes were analyzed using BLAST against the nr protein database (http://www.ncbi.nlm.nih.gov/). Using the same method, we identified 243 C2H2-ZF protein genes in *Drosophila melanogaster*, 290 genes in *Danaus plexippus*, 107 genes in *C. elegans*, 673 genes in *M. musculus*, and 1,082 genes in *H. sapiens* (Fig. 1). The information of those genes was list in Table S2.

**Figure 2:**
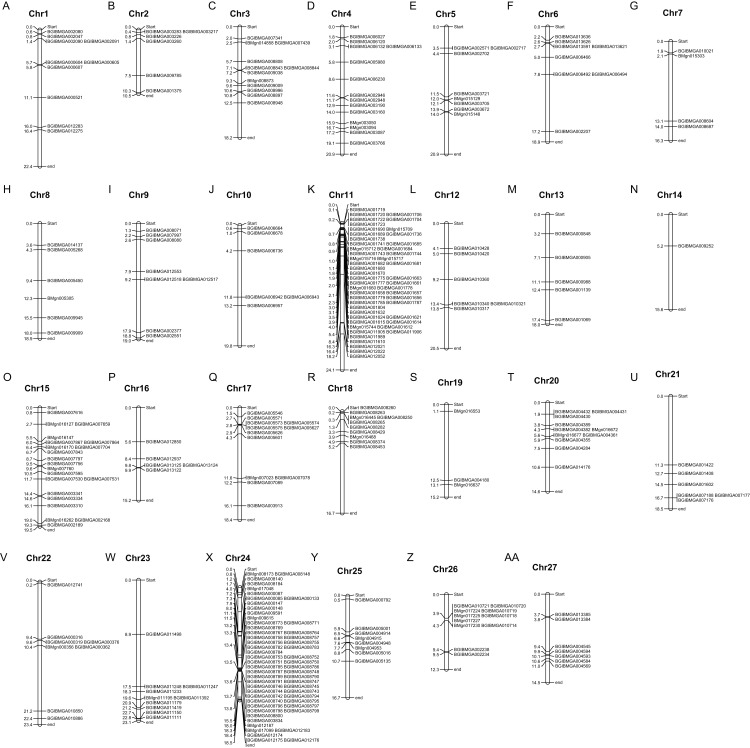
Chromosomal distribution of *BmZNF* genes. (A–AA) The distribution map of *BmZNF* genes on silkworm chromosomes was drawn by MapChart software. The abbreviation chr represents a chromosome. The distance unit on chromosomes is MB (mega bases).

Based on the genome assembly for the silkworm, we constructed a silkworm zinc finger C2H2-type protein gene distribution map. The members of the *BmZNF* gene family were widely and unevenly distributed on the chromosomes. As shown in Fig. 2, 312 of the 327 identified C2H2-type zinc finger protein genes were assigned to chromosomes 1–27 of the silkworm, and there was significant clustering on chromosomes 11 and 24. Chromosome 11 is about 24.1 mega bases (MB) in size, containing 50 ZNF genes, and chromosome 24 is about 18.5 MB, with 56 ZNF genes. The *BmZNF* genes on these two chromosomes account for about 1/3 of the total.

### PPIs domain analysis and phylogenetic tree of the BmZNF family

**Figure 3:**
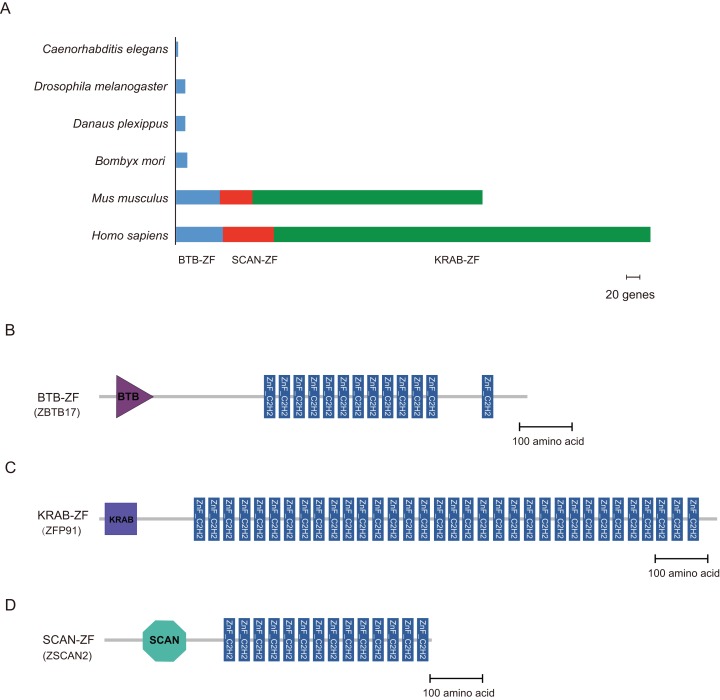
Distribution of BTB-ZF, KRAB-ZF, and SCAN-ZF proteins in the different species. (A) Representation of BTB-ZF, KRAB-ZF, and SCAN-ZF proteins in selected sequenced genomes. Six species were represented, and each type of protein architecture was showed as bar segments. (B–D) We selected three representative proteins, (ZBTB17, ZFP91, and ZSCAN2), of the human, which are corresponding BTB-ZF, KRAB-ZF, and SCAN-ZF, respectively. The domains structure of those proteins is represented.

Cys2-His2 zinc finger proteins often contain some protein-protein interaction (PPI) domains; for example, BTB (Broad-Complex, Tramtrack, and Bric-a-brac) (Zollman et al., 1994), the Krüppel-associated box (KRAB) (Mannini et al., 2006), and SCAN (SRE-ZBP, CTfin51, AW-1, and Number 18 cDNA) domain (Schumacher et al., 2000) are found in higher vertebrates. We used the hmmscan program in the HMMER package to perform the domain analysis. The C2H2-type zinc finger proteins of *B. mori*, *Danaus plexippus*, *Drosophila melanogaster*, *C. elegans*, *M. musculus*, and *H. sapiens* were compared to a well-characterized protein domain family in the HMMs database (Pfam-A) (Sonnhammer, Eddy & Durbin, 1997). There were 16, 13, 13, 3, 58, and 63 C2H2-ZF proteins containing the BTB domain (BTB-ZF-C2H2) in *B. mori*, *Danaus plexippus*, *Drosophila melanogaster*, *C. elegans*, *M. musculus*, and *H. sapiens*, respectively. In addition, the majority of C2H2-ZF family proteins are constructed of tandem zinc finger domains. Furthermore, KRAB- and SCAN-containing C2H2-ZF proteins were not found in any of the three insects and worm (Table S3). However, there were 497 and 303 ZNF proteins containing the KRAB domain in *H. sapiens* and *M. musculus*, respectively. And, the ZNF proteins containing the SCAN domain were found 67 in *H. sapiens* and 44 in *M. musculus*, respectively (Fig. 3).

In order to analyze the derivation of C2H2-type zinc finger proteins in the silkworm, we used the complete amino acid sequences predicted by the identified silkworm C2H2-type zinc finger proteins to generate a phylogenetic tree of BmZNF protein genes via the neighbor-joining method (Saitou & Nei, 1987). The 327 genes were categorized into three main grouping clades; the clades II and III had two subclades. The five groups (I, II a, II b, III a, and III b) contained 17, 78, 61, 57, and 114 proteins, respectively (Fig. S2).

### Tissue expression profile of BmZNF protein genes

**Figure 4:**
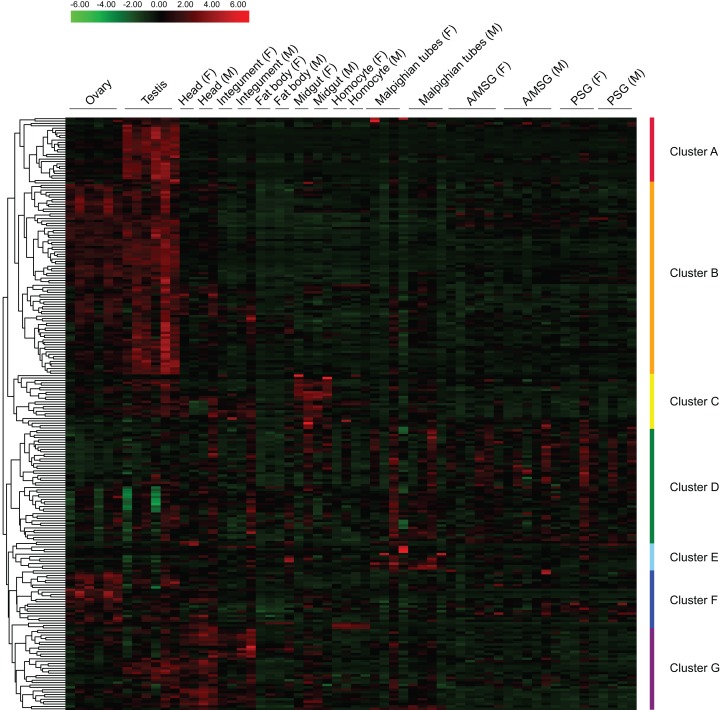
Expression profiling of *BmZNF* genes in multiple tissues of the silkworm. Each tissue sample was analyzed using at least two biological repeats. The heat map was constructed using the Pheatmap program of the R package. Clusters A–G are indicated by different colors. A/MSG, anterior/median silk gland; PSG, posterior silk gland; F, female; M, male. The expression profiling of *BmZNF* from the fourth day of the fifth instar to the adult stage.

Based on reported microarray data for the silkworm (Xia et al., 2007), we investigated the expression of BmZNF protein genes in multiple tissues of silkworm larvae on day 3 of the fifth instar. We found that 249 of the 327 *BmZNF* genes were expressed in at least one larval tissue during this stage. Notably, the genes of cluster A were highly expressed in testis, and those of cluster F were highly expressed in ovary. In addition, the *BmZNF* genes of cluster B were specifically expressed in both ovary and testis. The genes of cluster C were highly expressed in the midgut of silkworm larvae. The genes in cluster D had relatively high expression levels in the silk gland. The genes of cluster E were highly expressed in the Malpighian tubules. Moreover, the genes of cluster G were highly expressed in the head, integument and testis of silkworms (Fig. 4). The results showed that C2H2-type zinc finger protein genes of the silkworm appeared to have significant tissue-specific expression. This suggests that *BmZNF* genes assume regulatory functions in the development of different tissues.

### Developmental expression profile of BmZNF protein genes

**Figure 5:**
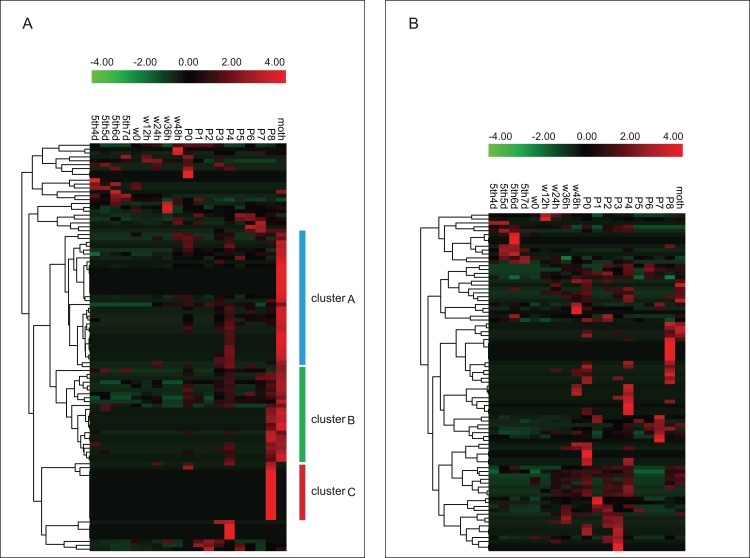
Expression profiling of *BmZNF* genes during development stages of the silkworm. (A) A total of 105 *BmZNF* genes are expressed in females. The clusters A, B and C are genes that are highly expressed in the late pupal stage and the adult stage (B) 87 *BmZNF* genes are expressed in males.

Using the metamorphosis microarray data of the silkworm from the later larval to the adult stages, the developmental expression profile of the silkworm C2H2-type zinc finger protein genes was surveyed. There were 105 and 87 BmZNF protein genes that were expressed during silkworm metamorphosis in females and males, respectively (Table S4). Notably, the genes of cluster A were highly expressed on day 1 of the adult moth; cluster B genes were highly expressed at 8 days of the pupal phase and on day 1 of the adult moth, and the genes in cluster C were specifically expressed at 8 days of the pupal phase in female silkworms (Fig. 5A). Interestingly, the above expression pattern of *BmZNF* genes was significantly different in males (Fig. 5B).

**Figure 6:**
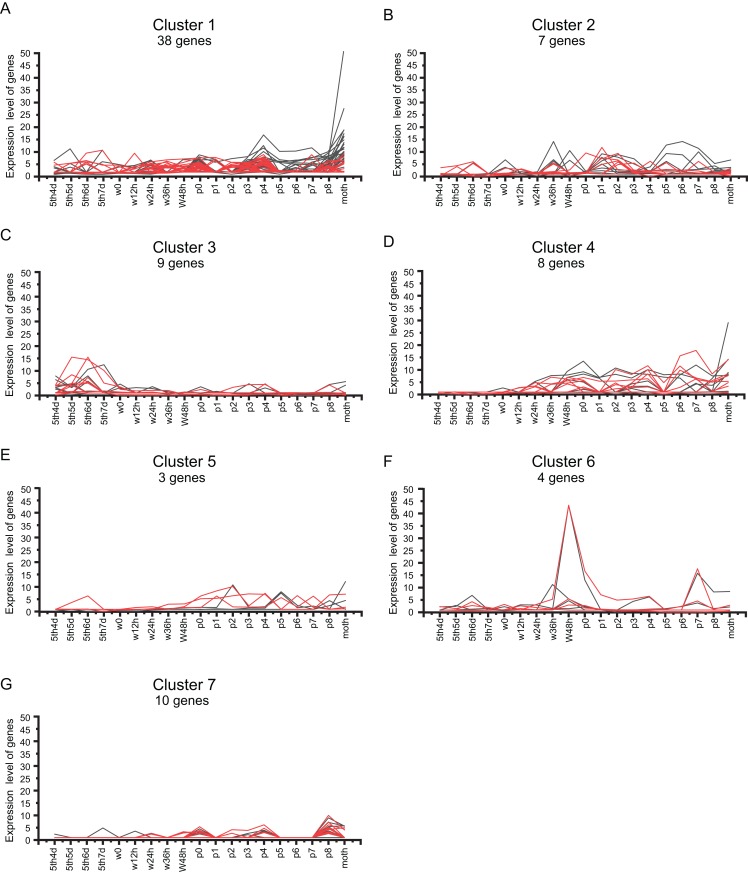
Cluster analysis of *BmZNF* gene expression in both sexes. (A–G) The *K*-mean/*K*-means cluster method was used to analyze the shared genes that are expressed in males and females. Clusters 1–7 represent different expression patterns of *BmZNF* genes. Red indicates male, black indicates female.

We analyzed the *BmZNF* genes that were expressed in both sexes. As shown in Fig. 6, 79 overlapping genes in females and males were grouped into seven clusters by *K*-means and *K*-medians clustering analysis using the MultiExperiment Viewer 4.90 software (Saeed et al., 2003). The genes of cluster 1 had a wavelike expression pattern in the larval to adult stages, and these genes were up-regulated in the adult. The genes of cluster 2 had a similar fluctuating expression pattern as in cluster 1, but these genes were not remarkable up-regulated in the adult moth stage. Notably, the genes of cluster 3 were mainly up-regulation expressed during the later larval stages and had low expression levels from the wandering larval to adult stages. Interestingly, the genes of cluster 4 were up-regulation expressed from wandering larvae to adult, but there was a lower expression in the early larval stages. The genes of cluster 5 were up-regulation expressed from the beginning of pupation to the adult stage, with a low expression at the early larval and wandering larval stages, except for sw01846 (*BmZNF294*) which was up-regulation expressed at day 5 of the fifth instar to day 6 of fifth instar larvae in males. Furthermore, the genes of cluster 6 displayed peak expression at 48 h after the beginning of the wandering larval stage (W48h) and on day 7 of the pupal stage (P7), and the genes of cluster 7 displayed peak expression on P0 and P8. These times were during key periods of pupation and eclosion. The 79 overlapping genes exhibited seven different expression patterns. The expression patterns suggest that the gene groups may participate in different phases of metamorphosis. For example, genes of cluster 3 were expressed at the later larval stages. The genes of cluster 4 mainly contributed to pupation and eclosion. The genes of clusters 6 and 7 may function as activating factors in critical developmental stages of metamorphosis.

### Analysis of *BmZNF* gene expression in ovarian developmental period

**Figure 7:**
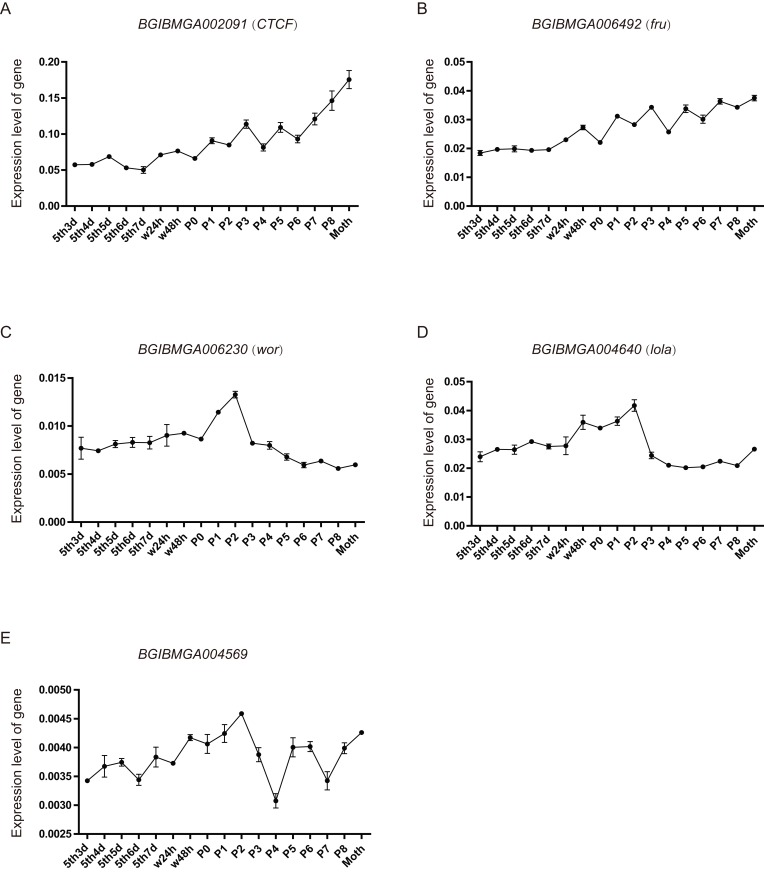
The expression pattern of *ZNF* gene in ovary of silkworm. (A–E) The five genes, *BGIBMGA002091* (*CTCF*), *BGIBMGA006492* (*fru*), *BGIBMGA006230* (*wor*), *BGIBMGA004640* (*lola*), and *BGIBMGA004569*, were detected the expression level in silkworm ovary, during day 3 of the fifth instar to adult, by qRT-PCR.

From the above analysis, found that many *BmZNF* genes were specifically significantly up-regulated expression in females. We speculate that their up-regulated expression may be involved in the regulation of female reproduction. Five of these genes were selected, and their expression levels were investigated in the ovarian tissues of silkworm from day 3 of 5th instar to adult, by qRT-PCR experiment. The *BGIBMGA002091* (*CTCF*) and *BGIBMGA006492* (*fru*) gene shared a similar rising expression pattern, during the development of ovary. Moreover, the expression level of *BGIBMGA002091* (*CTCF*) is significantly higher than that of *BGIBMGA006492* (*fru*). The *BGIBMGA006230* (*wor*) and BGIBMGA004640 (*lola*) gene have a saddle-shaped expression pattern in ovary, which were up-regulation expressed from the P0 to P3. The *BGIBMGA004569* gene have a wavelike expression pattern, and its expression level is the lowest among the five genes (Fig. 7).

## Discussion

The C2H2-ZF proteins are the most common and diverse type of TFs. In this study, we identified 327, 290, 243, 107, 673, and 1,082 C2H2-type zinc finger protein genes, accounting for approximately 1.9%, 1.9%, 1.6%, 0.4%, 2.6%, and 3.2% of the total gene number in *B. mori*, *Danaus plexippus*, *Drosophila melanogaster*, *C. elegans*, *M. musculus*, and *H. sapiens*, respectively. This indicates that the ZNF protein genes occupy a large proportion of higher taxa’ genome, and with the increase of species complexity, the number of genes in the *ZNF* family is also increasing. Meanwhile, these genes show obvious differentiation. Even among insect species, more than 100 *ZNF* genes in *Drosophila* have no orthologous genes in *B. mori* or monarch butterflies (Table S5). Moreover, the zinc finger proteins often contain PPI domains besides the tandem zinc finger domain. We identified 16, 13, 13, 3, 58, and 63 zinc finger proteins containing the BTB domain (BTB-ZF) in *B. mori*, *Danaus plexippus*, *Drosophila melanogaster*, *C. elegans*, *M. musculus*, and *H. sapiens*, respectively. The C2H2-ZF proteins containing KRBA and SCAN are common in mammals but were not found in the three insects and worms. This implies that the BTB domain may be more primitive in evolution compared to the other two PPI domains. Furthermore, these C2H2 zinc finger proteins also harbor other DNA binding domains such as Homeobox (PF00046.28), HTH_psq (PF05225.15), GAGA (PF09237.10), and BolA (PF01722.17). This implies that the mechanism by which these proteins bind DNA sequences may be more complicated. In addition, the 243 C2H2-type zinc finger protein genes of *Drosophila melanogaster* produced 556 C2H2-ZF proteins due to the fact that most zinc finger protein genes have alternative splicing patterns. Nevertheless, due to incomplete protein data, the total number of proteins translated by C2H2-type zinc finger protein genes is unclear in *B. mori* and *Danaus plexippus*. However, those species have dozens more C2H2-ZF protein genes than *Drosophila melanogaster*; we may surmise that the silkworm and monarch produce more C2H2-ZF proteins than the fruit fly. Previous research has shown that alternative splicing patterns of genes function in different tissues or during different periods (Venables, Tazi & Juge, 2012). This makes the study of zinc finger proteins more complicated. In the future, studies concentrated on a particular period and tissue of the focal organism will be necessary to clarify the functions and evolution of C2H2-ZF protein genes.

The analysis of the tissue expression data shows that C2H2-ZF protein genes exhibit tissue-specific expression patterns in silkworm larvae. Interestingly, over 40% of the *BmZNF* genes were expressed in gonad tissue, in which 28 *BmZNF* genes were specific to the testis, 24 *BmZNF* genes were significantly up-regulated in ovary, and 80 *BmZNF* genes were highly expressed in both male and female gonad tissues. The *BGIBMGA007530* gene is orthologous to *tramtrack*, which plays an important role in regulation of the development of the compound eye (Xiong & Montell, 1993), nerves (Badenhorst, 2001), and trachea (Araujo, Cela & Llimargas, 2007) in fruit flies; a ubiquitous TF *CTCF* (*BGIBMGA002091*) that binds to insulators and domain boundaries (Filippova et al., 1996) was highly expressed in the gonads of both sexes. The *BGIBMGA008740* gene is orthologous to the grauzone (*grau*) gene that is expressed in the ovary, and the loss of its function can lead to infertility in *Drosophila*. The expression of this gene in the silkworm was specific to the testis. The *ovo* (*BGIBMGA000988*) gene is an important factor in female germline differentiation (Andrews et al., 2000; Xue et al., 2014). The *BGIBMGA008808* is orthologous to *bowl*, which contributes to regulation of limb development (Ibeas & Bray, 2003). The *BGIBMGA002702* is orthologous to *ken*, which is involved in genital formation in flies (Lukacsovich et al., 2003). The latter three genes were highly expressed in the ovary of the silkworm. These zinc finger proteins, which as mentioned above have been well researched in *Drosophila melanogaster*, have an important relationship with tissue development. Therefore, the other BmZNF protein genes that are highly expressed in the tissues may also play important roles in the regulation of development. This provides a reference for subsequent functional studies.

Period expression analysis of *BmZNF* genes from fifth-instar 4-day larva to the adult stage shows that some genes have differential expression patterns in the two sexes. These differentially expressed genes may be involved in the development of sexual dimorphism. In females, we found that a group of genes was highly expressed near the adult stage. The genes of cluster III were highly expressed at day 8 of the pupa, the last stage of pupation. For example, the *BGIBMGA013385* is orthologous to the *dati* gene of this cluster; loss function of this gene can cause rejection of male courtship, resulting in female functional sterility in *Drosophila* (Schinaman et al., 2014). The genes of cluster I including *BGIBMGA006492*, an ortholog of *fru* that contributes to courtship behavior (Manoli et al., 2005), were highly expressed at day 1 of the adult moth. Furthermore, the genes of cluster II were highly expressed at day 8 of the pupal stage and day 1 of the adult moth. The gene *BGIBMGA008800* (a paralogue of *BGIBMGA008740*) in this cluster is orthologous to the *grau* gene, of which dysfunction results in female sterility, meiosis II arrest, and egg activation defects (Chen et al., 2000). Based on these results, we can infer that these gene clusters may be related to mating, ovary development and egg laying in female moths.

In addition, we analyzed the genes expressed in both sexes. Cluster analysis of period expression was performed for 79 genes expressed in both sexes. We found that 45 *BmZNF* genes were expressed from the terminal larval stage to the adult stage, and 34 *BmZNF* genes were expressed at a specific stage. The different patterns of expression of these genes imply that they may perform different tasks during metamorphosis. For example, the genes of cluster 3 were notable for high expression at the later stages of larval development followed by low levels of expression. The *BGIBMGA002047* gene is orthologous to the *luna* gene of cluster 3, a gene that belongs to the Krüppel-like TF family. The *luna* gene plays an important role in *Drosophila* development; mutations of the gene can cause larval death (De Graeve et al., 2003). These genes from cluster 3 may be necessary for larval stage development. Combined with the observation that the genes of cluster 4 were expressed from the wandering larva to the adult stage and the lack of expression in the early larval stages, suggests that these genes may be crucial for pupation and eclosion. The gene *BGIBMGA008080* is orthologous to the *noc* gene in cluster 4, which plays an important role in wing and leg development in the fruit fly (Weihe et al., 2004). The information of genes mentioned above was list in Table 1.

**Table 1:** C2H2 type zinc finger encoding genes and the phenotypes associated with their mutation.

Silkworm symbol	Gene ID	Fly symbol	Phenotypes in fly
*BmZNF158*	*BGIBMGA007530*	*tramtrack*	Compound eye, nerves and trachea
*BmZNF4*	*BGIBMGA002091*	*CTCF*	Gonads
*BmZNF273*	*BGIBMGA008740*	*grau*	Female sterility
*BmZNF279*	*BGIBMGA008800*	*grau*	Female sterility
*BmZNF141*	*BGIBMGA000988*	*ovo*	Female germline differentiation
*BmZNF20*	*BGIBMGA008808*	*bowl*	Limb development
*BmZNF45*	*BGIBMGA002702*	*ken*	Genital
*BmZNF306*	*BGIBMGA013385*	*dati*	Courtship
*BmZNF56*	*BGIBMGA006492*	*fru*	Courtship
*BmZNF2*	*BGIBMGA002047*	*luna*	Larval death
*BmZNF71*	*BGIBMGA008080*	*noc*	Wing and leg
*BmZNF34*	*BGIBMGA006230*	*wor*	Neurogenesis
*BmZNF325*	*BGIBMGA004640*	*lola*	Lethal

The analysis of period expression identifies the time when these *BmZNF* genes are active and provides a reference for future research on the functions of these genes. Five interesting genes were selected for investigate their expression in the ovaries of 5th instar larvae and adults. The qRT-PCR experiment indicated that the five genes, which up-regulated in females according to microarray data, were expressed in ovaries. This further suggests that these genes may regulate female ovarian development, in silkworm.

## Conclusions

This study is a comprehensive identification and expression analysis of C2H2 type zinc finger proteins in the silkworm *B. mori*. The results serve as a new resource for studying zinc finger protein TFs that are involved in the development of the silkworm and other species of Lepidoptera. The gonad and metamorphosis associated genes analyzed by our screening can provide references for further functional research of these genes.

## Supplemental Information

10.7717/peerj.7222/supp-1 Supplemental Information 1 Schematic representation of the C2H2-ZF protein–DNA interaction interface. Amino acid residues of the zinc fingers 1 and 2 are numbered according to their relative position from the start of the alpha helical domain, with -1 denoting the first residue before the helix. Bases N1, N2, N3, N4, N5, N6 and N7 are numbered sequentially from 5′ to 3′of the primary DNA strand, and the complementary bases are primed. The conducting of the primary strand is shown with solid arrows, and the conducting of the complementary strand is shown with dashed arrows.

10.7717/peerj.7222/supp-2 Supplemental Information 2 Phylogenetic tree of *BmZNF* genes. A neighbor-joining phylogenetic tree of the complete amino acid sequence of *BmZNF* proteins was constructed using the MUSCLE program and visualized using the MEGA7 software. Grouping clades are indicated by different colors.

10.7717/peerj.7222/supp-3 Supplemental Information 3 Primers used in this study.

10.7717/peerj.7222/supp-4 Supplemental Information 4 Complete list of identified C2H2-type zinc finger protein genes. We list all the C2H2-type zinc finger protein genes that were identified in this paper.

10.7717/peerj.7222/supp-5 Supplemental Information 5 Domain scanning of C2H2-type zinc finger genes. The identified domain of the C2H2-type zinc finger proteins, and a list of C2H2-ZF proteins containing the BTB domain.

10.7717/peerj.7222/supp-6 Supplemental Information 6 Microarray base expression data of silkworm C2H2-type zinc finger protein genes. The microarray data of *BmZNF* genes and list of clustering genes.

10.7717/peerj.7222/supp-7 Supplemental Information 7 Orthologous gene analysis. The orthologous genes of *Drosophila melanogaster*, *Bombyx mori* and *Danaus plexippus* are listed.

## Additional Information and Declarations

### Data Availability

The following information was supplied regarding data availability: Raw data is available in the Supplemental Files.
