# Supplementary figures and images for: Genome-wide identification and expression profiling of the C2H2-type zinc finger protein genes in the silkworm Bombyx mori
Source: PeerJ. 2019 Jul 5;7:e7222. doi: 10.7717/peerj.7222 (PMC6613534; doi:10.7717/peerj.7222)

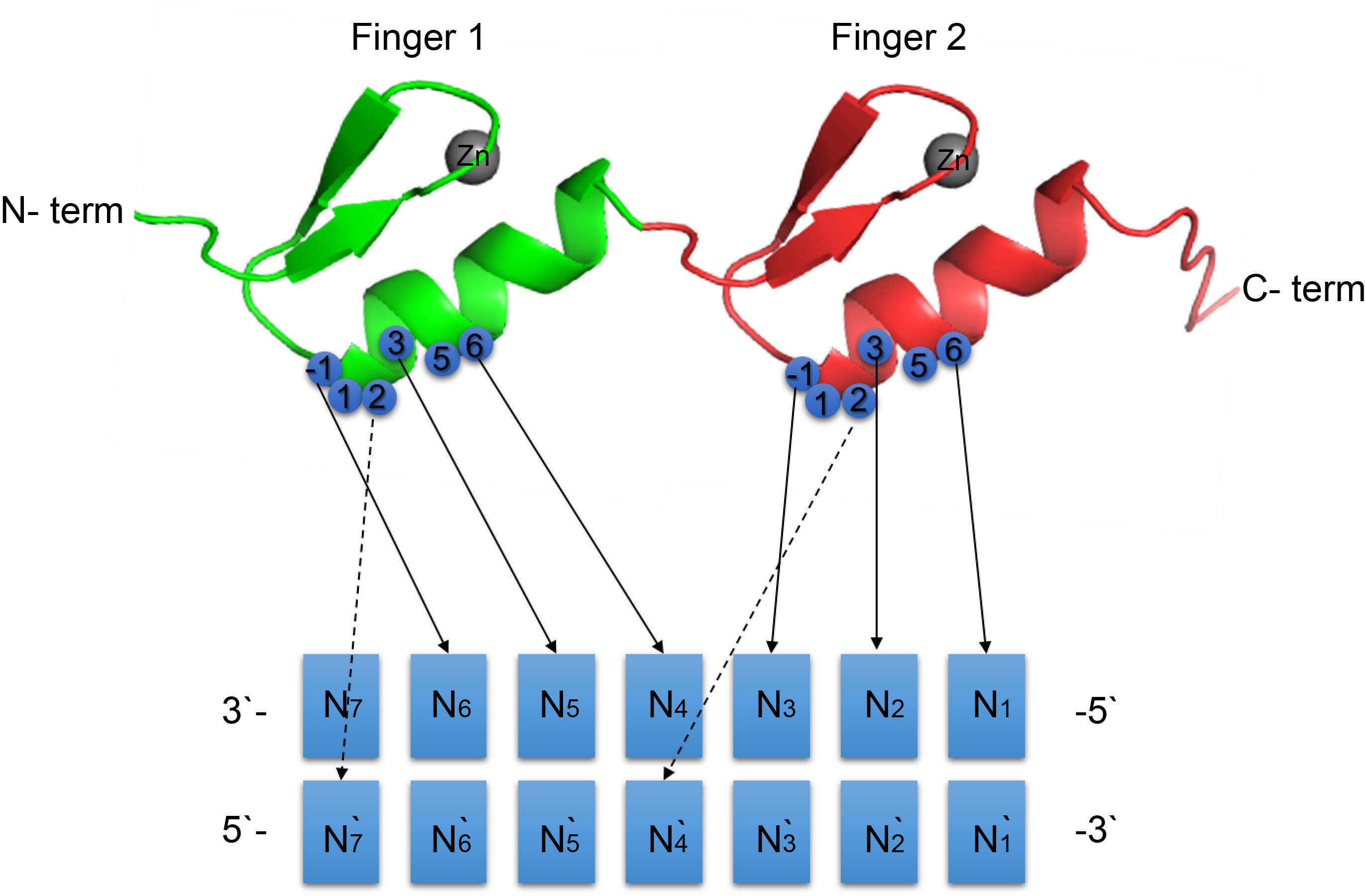

Supplement: Supplemental Information 1 — Amino acid residues of the zinc fingers 1 and 2 are numbered according to their relative position from the start of the alpha helical domain, with -1 denoting the first residue before the helix. Bases N1, N2, N3, N4, N5, N6 and N7 are numbered sequentially from 5′ to 3′of the primary DNA strand, and the complementary bases are primed. The conducting of the primary strand is shown with solid arrows, and the conducting of the complementary strand is shown with dashed arrows. [file peerj-07-7222-s001.jpg]

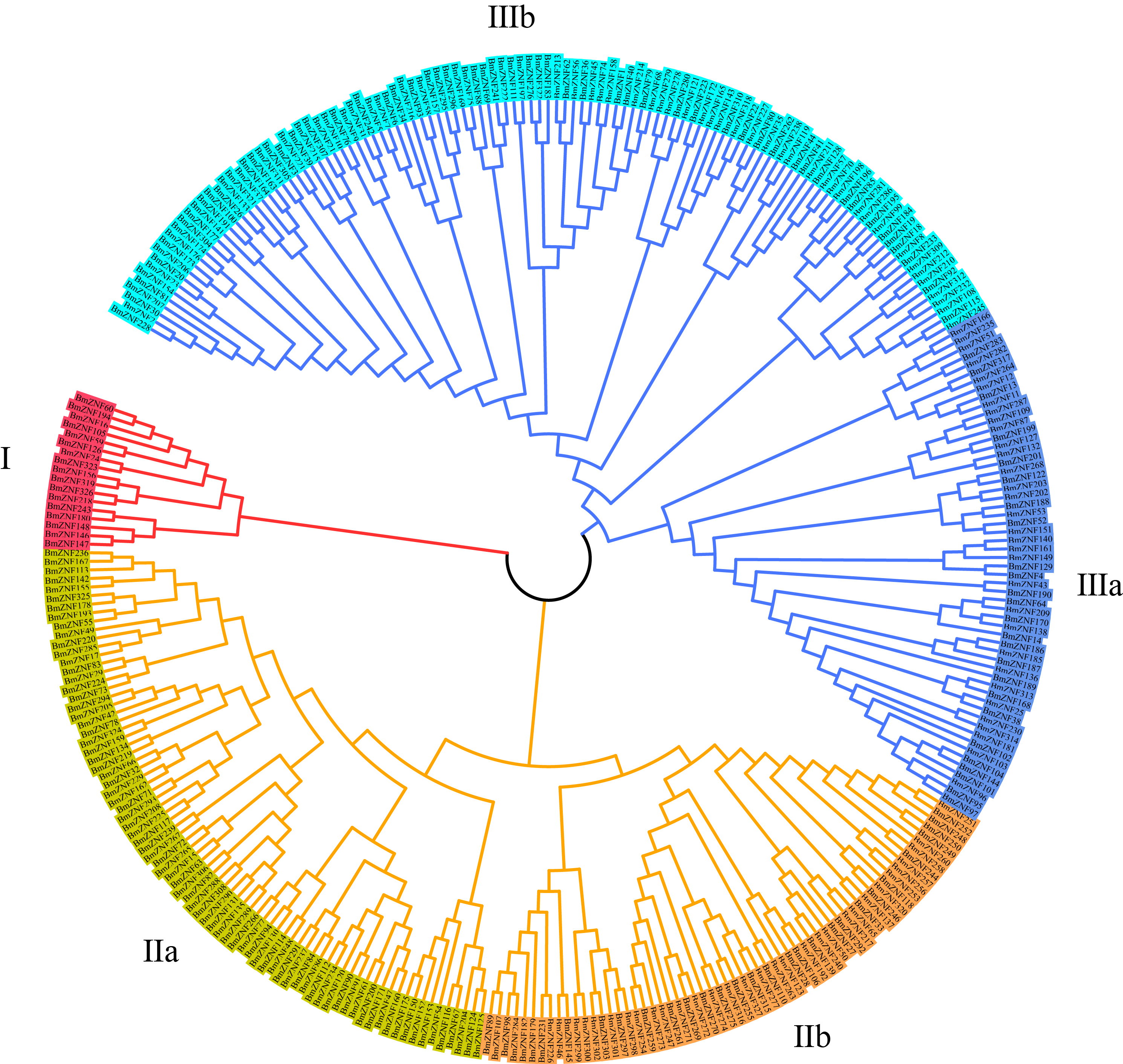

Supplement: Supplemental Information 2 — A neighbor-joining phylogenetic tree of the complete amino acid sequence of BmZNF proteins was constructed using the MUSCLE program and visualized using the MEGA7 software. Grouping clades are indicated by different colors. [file peerj-07-7222-s002.jpg]
