# Supplementary material for: Genome-wide identification and expression profiling of the C2H2-type zinc finger protein genes in the silkworm Bombyx mori
Source: PeerJ. 2019 Jul 5;7:e7222. doi: 10.7717/peerj.7222 (PMC6613534; doi:10.7717/peerj.7222)
Supplement: Supplemental Information 3 [file peerj-07-7222-s003.xlsx]

| Name | Symbol  in *Dm* | Forward | Reverse |
| --- | --- | --- | --- |
| *BGIBMGA002091* | *CTCF* | TTTGTTCGTATTCAAGCCACAG | TTTTCGTGTGTGTGCTTATAGC |
| *BGIBMGA006492* | *fru* | GGTGATAATTTCGGAGTTGACG | GACGAGATTTTCAACGTTCGAA |
| *BGIBMGA006230* | *wor* | GAAACTATCCGAGCAAAGCTTC | CGAATACAATGTCGGCATGTAA |
| *BGIBMGA004640* | *lola* | CATGGAGAGATCTCAAGACGAA | GAAAAGGAGTTTTTGCGTATGC |
| *BGIBMGA004569* |  | TCGAGTGCGACAAATTAAAGTG | GATGTTCAGGTGTATCCGGTTC |
| *BGIBMGA003186* | *eIF4A* | TTCGTACTGCTCTTCTCGT | CAAAGTTGATAGCAATTCCCT |

Primers used in this study
